# Expression of *Paracoccidioides brasiliensis AMY1* in a *Histoplasma capsulatum amy1* Mutant, Relates an α-(1,4)-Amylase to Cell Wall α-(1,3)-Glucan Synthesis

**DOI:** 10.1371/journal.pone.0050201

**Published:** 2012-11-20

**Authors:** Emma Camacho, Victoria E. Sepulveda, William E. Goldman, Gioconda San-Blas, Gustavo A. Niño-Vega

**Affiliations:** 1 Centro de Microbiología y Biología Celular, Instituto Venezolano de Investigaciones Científicas, Caracas, Venezuela; 2 Department of Microbiology and Immunology, University of North Carolina at Chapel Hill, Chapel Hill, North Carolina, United States of America; 3 Department of Molecular Microbiology, Washington University in St. Louis, St. Louis, Missouri, United States of America; Instituto de Salud Carlos III, Spain

## Abstract

In the cell walls of the pathogenic yeast phases of *Paracoccidioides brasiliensis, Blastomyces dermatitidis* and *Histoplasma capsulatum*, the outer α-(1,3)-glucan layer behaves as a virulence factor. In *H. capsulatum*, an α-(1,4)-amylase gene (*AMY1*) is essential for the synthesis of this polysaccharide, hence related to virulence. An orthologous gene to *H. capsulatum AMY1* was identified in *P. brasiliensis* and also labeled *AMY1*. *P. brasiliensis AMY1* transcriptional levels were increased during the yeast phase, which correlates with the presence of α-(1,3)-glucan as the major yeast cell wall polysaccharide. Complementation of a *H. capsulatum amy1* mutant strain with *P. brasiliensis AMY1*, suggests that *P. brasiliensis* Amy1p may play a role in the synthesis of cell wall α-(1,3)-glucan. To study some biochemical properties of *P. brasiliensis* Amy1p, the enzyme was overexpressed, purified and studied its activity profile with starch and amylopeptin. It showed a relatively higher hydrolyzing activity on amylopeptin than starch, producing oligosaccharides from 4 to 5 glucose residues. Our findings show that *P. brasiliensis* Amy1p produces maltooligosaccharides which may act as a primer molecule for the fungal cell wall α-(1,3)-glucan biosynthesis by Ags1p.

## Introduction

Paracoccidioidomycosis (PCM) is a human systemic mycosis restricted to Latin America, particularly Brazil, Colombia and Venezuela [1]. It is known to be caused by four cryptic species: S1, PS2 and PS3 [2], from the *Paracoccidioides brasiliensis* complex and *Paracoccidioides lutzii* (originally called Pb01-like) [3]. These species are thermo-dimorphic fungal pathogens that grow as mycelium under saprobic conditions (mycelial phase, M) at 23°C or as pathogenic yeast-like cells (yeast phase, Y) at 37°C. PCM mainly affects male rural workers whose occupation requires a close contact with the soil. Infection is thought to occur when conidia or hyphal fragments present in the environment are inhaled into the lungs, where they undergo a morphologic transition into yeast cells and grow in the lung parenchyma [4].

α-(1,3)-Glucan is a cell wall component of most fungal respiratory pathogens [5]–[9]. In *P. brasiliensis* it is found as the outermost layer of the Y cell wall [9]. The final step of its synthesis is associated with a single enzyme, α-1,3-glucan synthase (Ags1p), in *P. brasiliensis* [10], and *Histoplasma capsulatum* [11]. α-1,3-Glucan is absent in the mycelial phase and is correlated with virulence in *P. brasiliensis* [1], [12] and other closely related fungi such as *H. capsulatum* [11] and *Blastomyces dermatitidis* [6]. Sequence analysis of *P. brasiliensis* Ags1p revealed a common structure to other fungal Ags (Mok) proteins belonging to *Schizosaccharomyces pombe* and *Aspergillus niger* [10], [13], [14], [15]; they are composed of five domains from the N-terminal to the C-terminal ends, including a signal peptide, an extracellular α-amylase homology domain, a single transmembrane domain, an intracellular glycosyl-transferase domain and a multiple-spanning transmembrane domain [10]. In *S. pombe* it has been proposed that during vegetative growth, the glycosyl-transferase intracellular domain of Ags1p (Mok1p) is involved in the synthesis of single, linear α-glucan chains, each one consisting of approximately 120 α-(1,3)-linked glucose residues and some α-(1,4)-linked glucose residues at the reducing end. The extracellular α-amylase homology domain has been proposed to act as a transglycosylase coupling two α-glucan chains that are extruded to the periplasmic space through the multiple-spanning transmembrane domain. The final polysaccharide is a single population of linear glucose polymers composed of two interconnected linear chains [16]. Unlike the vegetative growth of *S. pombe*, in *P. brasiliensis* Y cells, α-(1,3)-glucan consists of a long linear chain of α-(1,3)-linked glucose units, occasionally branched by a single glucose moiety joined to the main chain by α-(1,4) linkages [10], a structure similar to the one produced by Mok12 and Mok13 during the *S. pombe* sporulation process [15].

The α-amylase superfamily comprises a large variety of enzymes with different activities and substrate specificities that are active on α-glucosidic bonds. Based on sequence similarity, members of this superfamily are divided into glycoside hydrolase (GH) families GH13, GH70 and GH77 [17]. The tertiary structure of these enzymes is characterized by a (β/α)_8_ barrel containing four highly conserved amino acid regions that form the catalytic site [18]. All family members hydrolyse and/or transglycosylate α-glucosidic linkages via a double displacement mechanism of catalysis [19]. Recent studies have demonstrated that different fungal GH13 enzymes might be associated with cell wall α-(1,3)-glucan production and/or modification, rather than with starch degradation. Among them, *S. pombe* Aah3p and *A. niger* AgtA, both glycosylphosphatidylinositol (GPI)-anchored proteins, are two novel types of GH13 family homologues that play a role in the integrity of the fungal cell wall. Disruption of *Aah3* causes an aberrant morphology of the cells, highly sensitive to cell wall-degrading enzymes [20]. The *agt* genes in aspergilli (*A. niger*, *A. nidulans*, *A. oryzae* and *A. fumigatus*) cluster with α-glucan synthase genes and others [21]. AgtA has 4-α-glucanotransferase activity on maltooligosaccharides; the disruption of the gene that encodes it also induces a similar aberrant phenotype in cell shape [22]. A second type of GH13 enzyme with a role in cell wall formation is *H. capsulatum* Amy1p, a putative intracellular α-amylase highly homologous to another GH13 fungal α-amylase, *A. niger* AmyD. Amy1p is essential for the synthesis of cell wall α-(1,3)-glucan and expression of virulence in *H. capsulatum* [23], while *A. niger* AmyD has a relatively low hydrolyzing activity on starch (2.2 U mg^−1^) which mainly leads to the production of maltotriose [24]. Due to the genomic arrangement among *agt* and *ags* genes previously described in aspergilli, it has been suggested that GH13 enzymes might play a role in a common metabolic path, perhaps α-(1,3)-glucan synthesis [21], [25].

In the present work, we aimed to test *P. brasiliensis AMY1* functionality by expressing it in a *H. capsulatum amy1* mutant strain and also by purifying the protein and analyzing its enzymatic activity. Our findings indicated that *P. brasiliensis AMY1* successfully complemented the *H. capsulatum amy1* mutant, and that *P. brasiliensis* Amy1p, also homologous to Amy1p and AmyD from *H. capsulatum* and *A. niger*, respectively, generates short oligosaccharides that might act as primers at the very first step of α-(1,3)-glucan production.

## Methods

### Strains, Media and Growth Conditions

All fungal strains and plasmids used in this study are listed in Table 1. *P. brasiliensis* strain IVIC Pb73 (ATCC 32071) was maintained by monthly subculture on YPG (0.5% (w/v) yeast extract, 0.5% (w/v) bactopeptone, 1.5% (w/v) glucose) agar slants. For *P. brasiliensis AMY1* gene expression, total RNA was isolated from mycelium (M) and yeast (Y) cells grown at 23°C or 37°C for 3 days, respectively. *H. capsulatum* strain backgrounds used in this study were obtained from the chemotype II strain G186A (ATCC 26029). They were grown in HMM medium (solid or liquid) at 37°C with 95% air-5% CO_2_ as previously described [26]. HMM consists of F-12 nutrient mixture with L-glutamine and phenol red but without sodium bicarbonate (Invitrogen) supplemented with the following (per liter): 18.2 g of glucose, 1.0 g of glutamic acid, 84 mg of cystine, and 5.96 g of N-2-hydroxyethylpiperazine-N’-2-ethanesulfonic acid adjusted to pH 7.5. HMM solid medium contained 0.8% agarose (SeaKem ME grade) and 25 mM FeSO_4_. For non-selective growth of *ura5* mutants, HMM was supplemented with 100 µg uracil ml^−1^ (Sigma). *Escherichia coli* QIAGEN EZ Competent Cells (Qiagen), were used for propagation of plasmids and cloning experiments, and were grown in Luria-Bertani (LB) medium supplemented with 100 µg ampicillin ml^−1^. *E. coli* M15[pREP4] (Qiagen) was used for protein production and purification, and grown in LB medium supplemented with 100 µg ampicillin ml^−1^ and 25 µg kanamycin ml^−1^.

**Table 1:** Fungal strains and plasmids used in this study.

Fungal strains	Genotype or description	Source or reference
*P. brasiliensis* IVIC Pb73	Phylogenetic group PS3†	ATCC 32071
*H. capsulatum* WU8	G186A *ura5-Δ32*	[23]
*H. capsulatum* WU11	G186A *ura5-Δ31 ags1-Δ3::hph*	[23]
*H. capsulatum* WU8	G186A *ura5-Δ31 amy1-Δ1::hph*	[23]
**Plasmids**		
pCR41	*PaURA5 P_CBP1_*	[11]
pEC87	*PaURA5 P_CBP1_-PbAMY1*	This study
pEC90	*PaURA5 P_AMY1_-PbAMY1*	This study
pQE30Xa	*E. coli* specific vector for high.level expression of N-terminal 6xHis-tagged proteins.	Qiagen, Germany
pQE30Xa-AMY	pQE30Xa containing *PbAMY1* cDNA	This study

*hph*, hygromycin phosphotransferase (hygromycin resistance).

PaURA5, Podospora anserina URA5 gene.

PbAMY1, P. brasiliensis AMY1 gene.

*P_CBP1_,* 889 bp upstream of the *H. capsulatum CBP1* gene.

*P_AMY1_,* 1.950 bp upstream of the *P. brasiliensis AMY1* gene.

† Defined by [2].

### Isolation and Sequencing of *P. brasiliensis AMY1* Gene

Primers used in this study are listed in Table 2. Taking advantage of degenerate primers (AMY1427-R and AMY1429-F) kindly provided by Dr. William E. Goldman (Department of Microbiology and Immunology, University of North Carolina at Chapel Hill, North Carolina, USA) and designed to identify an α-amylase in *H. capsulatum,* we amplified a 553 bp fragment that showed 77% homology to *H. capsulatum AMY1* (GenBank ABK62854). The sequence information allowed the design of PCR specific primers (AMY1-5 and AMY1-3 for 5′ and 3′ RACE, respectively) to obtain the full-length transcript. We performed 5′ and 3′ RACE (Clontech) using total RNA from *P. brasiliensis* IVIC Pb73 as a template and following the user’s manual. Full-length cDNA sequence was obtained by merging 5′- and 3′-end RACE fragments using the ContigExpress software from Vector NTI Suite (InforMax, Inc.). A new primer pair (AMY3sh and AMYSTOP) was designed at the very ends of the full-length cDNA and used for amplification of complete *AMY1* gene. Nucleotide sequencing was automated on ABI PRISM 3730XL DNA sequencer (Applied Biosystems) (Macrogen, Korea). We identified an ORF (open reading frame) of 2563 bp, interrupted by seven introns and encoding 535 amino acid residues (GenBank ABS11196.1).

**Table 2:** Primers used in this study.

Primers	Sequence (5′–3′)
AMY1427-R	Available on request to W.E.G.
AMY1429-F	Available on request to W.E.G.
AMY1-5	CGCACTTCTCTACATAATCGGCCGC
AMY1-3	GGGCTTTTCGTTTCCTGGTCGGGG
AMY3sh	GCGGGCATGTTAGTGGTTTCAGAT
AMYSTOP	CCAACGGCATCCACGGATAAAAGC
AmyRTqF2	GCATTCTTAGGCCGTCGTGTAATG
AmyRTqR	TTCTTATCTGCAGGGCCGTTACTG
18S S3	CGATTCCGGAGAGGGAGCC
18S AS3	CGTATCGGGATTGGGTAATTTGC
cDNA-AscF	AAAGGCGCGCCGCGGAATGG
cDNA-SpeR	GATGATAAGCACTAGTCGATCAGC
HcAMYpFKpn	GGTACCCGAATTTGCTTCTGGC
HcAMYpAsc	ATTGGCGCGCCACATGAGTGTCCC
Amy1pQE	TCAAAGGATTCCTGCGGAATGGC
Amy2pQE	GATGAGCTCTCGATCAGC

### *P. brasiliensis* Amy1p and Gene Analysis *in Silico*

**Figure 1:**
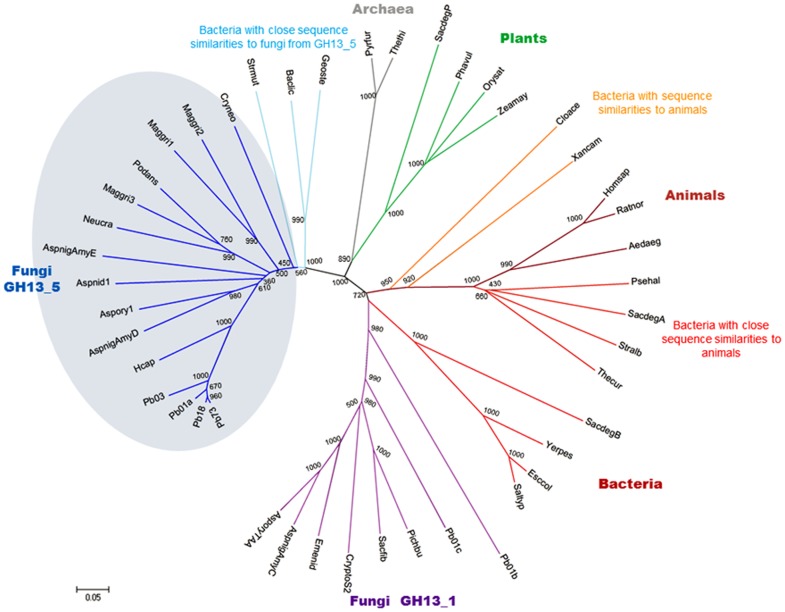
Phylogenetic tree of α-amylase proteins from representative taxa of the three domains of life. Abbreviations used and sources of the α-amylases are defined in Table S1. The tree is based on the alignment made in CLUSTAL W of the partial sequences encoding the (β/α)_8_ barrel. Two separate groups of fungal α-amylases, intracellular (circled, GH13_5) and extracellular (GH13_1), are distinctly noted. The branch length is proportional to the sequence divergence. Numbers along branches are bootstrap values (1000 replicates). The scale bar (bottom-left corner) indicates 0.05 amino acid substitutions per site.

A phylogenic analysis was done comparing Amy1p sequence against a set of 39 sequences encoding α-amylases retrieved from GenBank [27] and SWISS-PROT [28], and supplemented with 3 sequences from *Paracoccidioides lutzii* Pb01 (Pb01a, Pb01b and Pb01c) and 2 sequences from *P. brasiliensis* cryptic species (Pb03 and Pb18) (http://www.broadinstitute.org) (Figure 1, Table S1). The alignment strategy was based on the approach described by [29]. The tree was calculated by the neighbor-joining method [30] implemented in the MEGA software [31] using the final alignment including the gaps; the number of bootstrap trials used was 1000. The tree was displayed with the MEGA software [31].

### Quantitative PCR

Total RNA was extracted with TRIzol® (GIBCO) and treated with DNase using the TURBO DNA-free™ kit (Ambion). The RETROScript® kit (Ambion, Austin, TX, USA) was used for reverse transcription of mRNA. For quantitative PCR, 1/5th of the reverse-transcribed RNA was used as template to amplify *AMY1* with the primers AmyRTqF2 and AmyRTqR (Table 2); *18S* was used as the normalizer gene, with the help of primers 18S S3 and 18 AS3 (Table 2). Quantitative PCR was performed in triplicate on an iQ5 real-time PCR detection system, using the GoTaq® qPCR Master Mix kit (Promega Corporation). All Ct values were normalized to the Ct values of the standard gene and the relative expression levels were calculated using the 2^−ΔΔCT^ method [32]. Melting curve analysis showed that all PCR products corresponded to the targeted transcript (data not shown).

### Transformation of a *H. capsulatum amy1* Mutant with *P. brasiliensis AMY1* Gene

Constructions of telomeric plasmids carrying *P. brasiliensis AMY1* under either the *H. capsulatum CBP1* or *AMY1* promoters were done with plasmid pCR41 (empty vector), previously described by [11]. Primers cDNA-AscF and cDNA-SpeR (Table 2) were designed to amplify a 1611 bp fragment corresponding to *P. brasiliensis AMY1* coding sequence. The PCR product was digested with *Asc*I/*Spe*I and cloned into the pCR41 plasmid at the *Asc*I/*Spe*I sites, yielding plasmid pEC87, in which *P. brasiliensis AMY1* is driven by the *H. capsulatum CBP1* promoter. In order to assay *P. brasiliensis AMY1* expression under *H. capsulatum AMY1* promoter, primers HcAMYpFKpn and HcAMYpAsc (Table 2) were designed to amplify a 1950 bp fragment of the *H. capsulatum AMY1* promoter region. The PCR product was digested with *Kpn*I/*Asc*I and cloned into the pEC87 plasmid at the *Kpn*I/*Asc*I sites, removing the *CBP1* promoter and yielding plasmid pEC90. Transformation of *Histoplasma* with telomeric plasmids was performed [33] using 100–200 ng of linearized DNA and plating on HMM media. All experiments included *H. capsulatum* control strains transformed with plasmid pCR41, which complements uracil auxotrophy.

### Microscopy

For immunolocalization of cell wall α-(1,3)-glucan, yeast cells in stationary phase grown in HMM culture medium were washed three times with 1X PBS (0.8 g NaCl, 0.65 g Na_2_HPO_4_, 0.2 g KCl, 0.2 g KH_2_PO_4_, pH 7.3) and fixed in 4% w/v formaldehyde in PBS for 15 min at room temperature. For microscopic observation, 50 µl of yeast suspension were smeared onto polylysine-covered slides, air-dried, washed with PBS and stained with a mouse monoclonal IgM antibody that recognizes α-(1,3)-glucan (MOPC104E, M5750, Sigma). DAPI was added to the final wash to visualize DNA. The observation was carried out using a fluorescent microscope (Eclipse E600; Nikon, Tokyo, Japan) equipped with epifluorescence illumination and a UV-2A filter. In every case, the neutral density filter ND4 was used. Photographs of fluorescent images were taken with a digital camera (Nikon Coolpix 8700).

### *H. capsulatum* Yeast Cell Wall α-(1,3)-glucan Quantification

*Histoplasma* yeast cells grown to stationary phase in HMM liquid culture were collected by centrifugation, washed twice with autoclaved Milli-Q water and disrupted with acid-free glass beads (5×1 min with 1 min intervals, on ice). Cell wall material was collected by centrifugation at 8000 *g* for 15 min, frozen at −80°C and lyophilized. Yeast cell walls were biochemically fractionated using 1 M NaOH extraction at room temperature overnight, followed by centrifugation at 8000 *g* for 10 min, and acetic acid neutralization of the alkali-soluble fraction [34]. The α-(1,3)-glucan-containing fraction (alkali-soluble, acid-precipitable fraction) was subjected to acid hydrolysis with 1 M HCl, to quantify its glucose content by means of the anthrone reaction [35].

### Fourier Transformed Infrared (FTIR) Spectroscopy

Samples were prepared as KBr pellets. FTIR spectra were recorded from 4000 to 400 cm^−1^, using a Nicolet iS10 spectrometer (Thermo Fisher Scientific,Waltham, MA).

### Macrophage Culture and Virulence Assay

P388D1 mouse macrophage-like cells were cultured in F-12 medium (Gibco) +10% FBS (HyClone) or in HMM-M when coincubated with *Histoplasma* yeasts, as described previously [36]. *Histoplasma* virulence for P388D1 cells was determined as previously described [11], [37]. Briefly, monolayers of P388D1 cells were infected in triplicate with *Histoplasma* yeasts at a multiplicity of infection of 1∶3 (yeasts:macrophages) in 24 well plates and incubated at 37°C in 95% air-5% CO_2_. Fifty percent of the culture medium was replaced with fresh media every 3 days. Following 8 days of infection, the culture medium was removed and remaining macrophages lysed with a solution containing 10 mM Tris, 1 mM EDTA, and 0.05% SDS. PicoGreen double-stranded DNA quantification reagent (Molecular Probes) was used to measure the amount of macrophage DNA remaining in each well. Data represent results collected from three independent assays.

### Protein Production and Purification

For heterologous expression of *P. brasiliensis AMY1*, primers Amy1pQE and Amy2pQE (Table 2) were designed to amplify a fragment of 1700 bp corresponding to the *P. brasiliensis AMY1* coding sequence. The PCR product was digested with *Bam*HI/*Sac*I and cloned into the pQE30Xa expression vector (Qiagen) at the *Bam*HI/*Sac*I sites, yielding plasmid pQE30Xa-AMY. *E. coli* M15[pREP4] transformed with either pQE30Xa (empty vector) or pQE30Xa-AMY was grown in LB medium supplemented with 100 µg ampicillin ml^−1^ and 25 µg kanamycin ml^−1^ at 28°C until an OD_600_ of 0.4 was reached. Expression was induced by the addition of 1 mM IPTG and cultures were grown until OD_600_ was 0.8–1.0. Cells were harvested by centrifugation (10 min, 5000 *g*, 4°C) and washed with 50 mM Tris-HCl buffer (pH 8). Cell pellets were resuspended in binding buffer pH 7.2 (50 mM NaHPO_4_.H_2_O, 500 mM NaCl and 5 mM imidazole), lyzozyme (1 mg/ml) and protease inhibitor cocktail (1 ml/20 g wet weight) (P8849, Sigma-Aldrich), and then incubated for 30 min on ice. Cell-free extracts were produced by sonication of the resuspended cells (8×5 sec with 40 sec intervals, on ice) and subsequent centrifugation (20 min, 4°C, 5000 *g*). The cell lysate supernatant was incubated overnight with nickel-nitriloacetate (Ni-NTA) agarose (Qiagen) at 4°C. The mix was washed twice with wash buffer (50 mM NaHPO_4_.H_2_O, 500 mM NaCl, pH 7.2). His-tagged proteins were eluted from the bound resin by incubation for 40 min at 4°C with elution buffer (50 mM NaHPO_4_.H_2_O, 250 mM NaCl and 300 mM imidazole, pH 7.2). At each stage of protein purification, the amount of protein was measured by means of the Bradford method with reagents from Bio-Rad, and purity was checked by SDS-PAGE analysis [38]. After Ni-NTA purification, Amy1p was used in biochemical assays in Na-barbital buffer pH 5.0 (28.5 mM sodium acetate, 28.5 mM Na-barbital and 116 mM NaCl) for a maximum of 24 hours.

### Analysis of Enzyme Activity

In order to measure *P. brasiliensis* Amy1p hydrolysing activity the standard reaction conditions were as follows: the enzyme was incubated with 0.2% (w/v) potato starch (S-2630, Sigma) in Na-barbital buffer (pH 5.0) for 1 hour at 37°C. Reactions were performed in a total volume of 300 µl. After digestion, 25 µl samples (triplicates) were diluted in 25 µl Na-barbital buffer (pH 5.0) and subsequently used to determine reducing ends by means of the bicinchoninic acid method [3]. The amount of enzyme depended on the batch, with 1 U defined as the amount of enzyme producing 1 µmol of reducing ends min^−1^. In all assays, reactions with equal or higher amount of protein from *E. coli* M15[pREP4] transformed with the empty vector were included to check for background activity and as negative control. The optimum pH was determined by performing the standard reaction at pH values between 4.5 and 8.0 in Na-barbital buffer.

For thin layer chromatography (TLC) studies, 300 µl-enzymatic reactions were lyophilized and resuspended in 100 µl of autoclaved distilled water. A total of 5 µl of reaction product and others was spotted on a TLC plate (Silica gel 60 F_254_, EMD Chemicals) and after drying, the plate was run twice for 5 h (each time) in a small amount of running buffer (butanol/ethanol/distilled water, 5∶ 5: 3, v/v). After running, the plate was dried and sprayed with a permanganate staining [0.75% (w/v) KMnO_4_, 5% (w/v) K_2_CO_3_, 5% aqueous NaOH (0.625 g)] and developed for 30 min at 60°C. As reference patterns, 1.5 µl of chemically pure glucose, maltose and maltotriose (Sigma) were spotted on the same TLC plate along with the enzymatic reactions samples. Starch and amylopeptin, used in activity assays, were originated from potato (S-2630 and A-8515 Sigma, respectively).

Zymogram analysis was performed by running 5, 10 or 20 µg of Ni-NTA purified protein on SDS-PAGE gels containing 10% polyacrylamide and 0.3% (w/v) amylopectin azure (A4640, Sigma). The protein samples were not boiled to preserve enzymatic activity. After separation, the gel was treated as described previously [39]. Afterwards, the same gel was stained with Coomassie (Bio-Rad).

## Results

### *P. brasiliensis* Amy1p *in silico* Analysis and *AMY1* Expression

**Figure 2:**
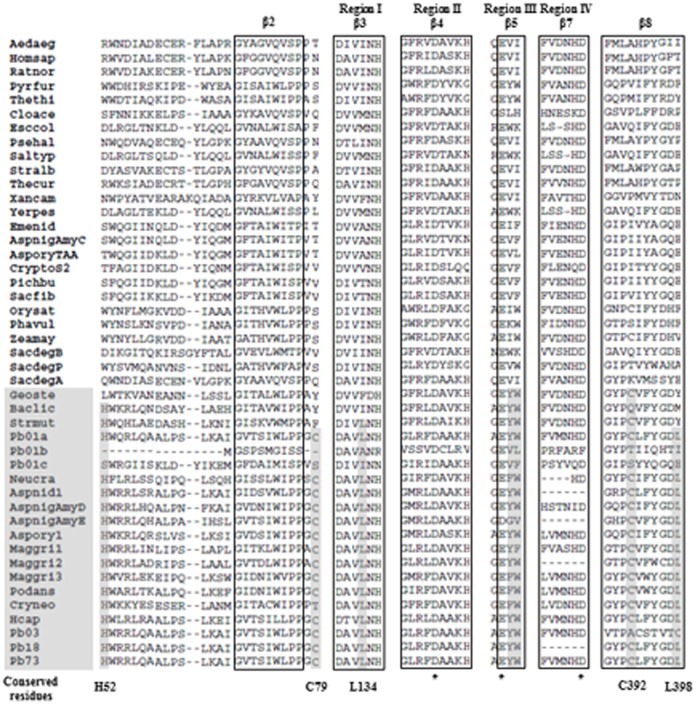
Partial alignment of α-amylase proteins used for construction of the phylogenetic tree. Highlighted in gray are the amino acid residues conserved among most intracellular fungal α-amylases and bacterial α-amylases that belong to subfamily GH13_5. An asterisk indicates the three amino acid residues involved in the catalytic site.

The deduced *P. brasiliensis* Amy1p amino acid sequence was 77 and 56% identical to *H. capsulatum* Amy1p (GenBank ABK62854) and *A. niger* AmyD (GenBank CAK37367.1), respectively. The Amy1p sequence, along with 5 sequences from *P. brasiliensis* complex and *P. lutzii* Pb01, were aligned with 39 α-amylases from representative taxa of the three domains of life: bacteria, archaea and eukarya. The phylogenetic tree (Figure 1) indicates that *P. brasiliensis* Amy1p groups with α-amylases from the GH13_5 subfamily. All of them, including *P. brasiliensis* Amy1p, are predicted to be intracellular enzymes according to the software SignalP 3.0 [40]. The sequence alignment allowed the identification of the three catalytic residues [a glutamic acid (E295) and two aspartic acids (D265 and D360) in *P. brasiliensis* Amy1p numbering] and the four highly conserved regions previously described in the primary sequence of α-amylases [41] (Figure 2). Lysine and histidine residues within the conserved region II (261_GLRFDAA**KH**), and associated with hydrolysis of α-(1,4) glycosidic bonds, were present. The alignment also showed features shared among most intracellular α-amylases and bacterial α-amylases of the liquefying type (GH13_5) (*Bacillus licheniformis*, *Geobacillus stearothermophilus* and *Streptococcus mutans*) [24]. Features include: (i) histidine (H52) and cysteine (C79), in the region flanking the β2 strand at the N-terminus and C-terminus, respectively; (ii) an invariant leucine residue prior to the conserved NH in conserved region I, at the end of the β3-strand region (131_DAV**L**NH); (iii) two aromatic residues following the catalytic glutamate proton donor in conserved region III (around strand β5) (294_AE**YW**K); and (iv) cysteine and leucine residues in the region covering the β8 strand (392_**C**LFYGD**L**) (Figure 2). Moreover, *in silico* analysis of *P. brasiliensis* Amy1p sequence identified a single motif for glycosylation and multiple sites for phosphorylation and myristoylation, suggestive of possible post-translational modifications.

**Figure 3:**
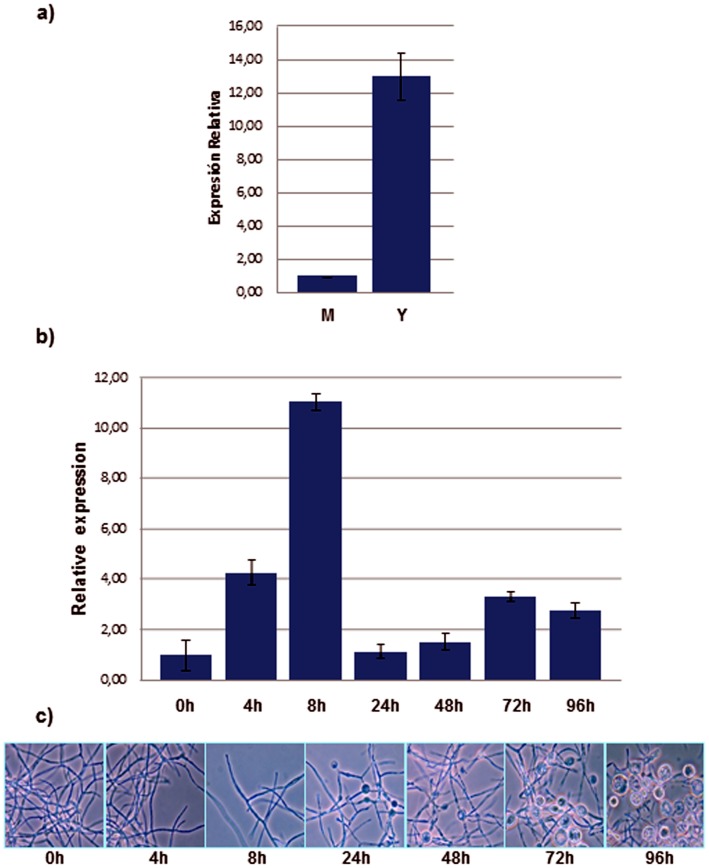
Expression analysis of *P. brasiliensis AMY1*. (**a**) Transcriptional levels of *P. brasiliensis AMY1* gene in the mycelial (M) and yeast (Y) phases by qRT-PCR. (**b**) Transcriptional level of *P. brasiliensis AMY1* during the mycelia to yeast dimorphic transition by qPCR. Transcript levels were normalized to the reference gene *18S* rRNA. Data represent two independent assays. Samples were assayed by triplicate. Error bars represent the standard deviation. (*) Mann-Whitney test between M and Y; *P*-value <0.005. (**c**) *P. brasiliensis* culture at each time point of the mycelia to yeast dimorphic transition.

qPCR expression analysis of *P. brasiliensis AMY1* showed that its transcriptional levels were almost 13 times higher during the yeast phase compared to the mycelial phase (Figure 3a). Mycelium to yeast transition was accompanied by an 11-fold increase in the transcriptional levels of *AMY1* at 8 hours into the transition, followed by a sharp decline nearly to the values measured at 0 hours. Later there was a gradual and slow increase in *AMY1* transcriptional levels until 72 hours (Figure 3b), suggesting a participation of *P. brasiliensis AMY1* at early time points of the mycelia to yeast transition and during the pathogenic phase.

### Complementation of *H. capsulatum Amy1* Mutant with *P. brasiliensis AMY1*

**Figure 4:**
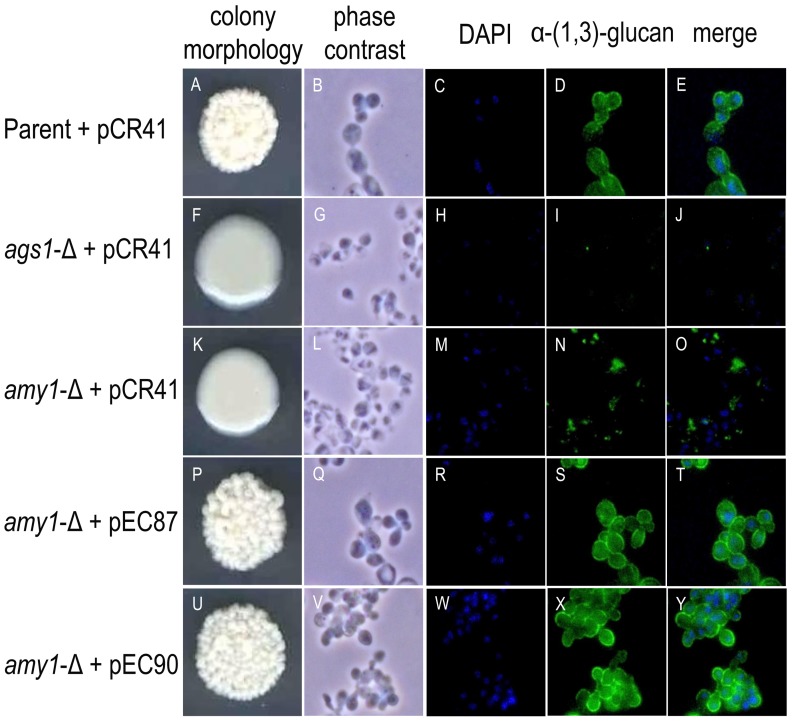
Colony morphology and α-(1,3)-glucan immunostaining. (**p**) and (**u**) *H. capsulatum amy1*-null mutant complemented with *P. brasiliensis AMY1* under the *CBP1* promoter (pEC87) or *AMY1* promoter (pEC90), respectively, is able to restore the rough phenotype exhibited by (**a**) the parental strain, as well as its α-(1,3)-glucan content and distribution. The smooth colony morphology correlates with loss of α-(1,3)-glucan from yeast cell walls as shown by immunofluorescence of (**f**) *ags1*-Δ mutant yeasts or (**k**) *amy1*-Δ mutant yeasts.

In *H. capsulatum* yeast cells, deletion of *AMY1* leads to a “smooth” colony morphology due to the markedly decreased levels of α-(1,3)-glucan [23]. To determine whether *P. brasiliensis* Amy1p has the same function as *H. capsulatum* Amy1p, the *P. brasiliensis AMY1* ORF was cloned into the telomeric plasmid pCR41. Two different promoters were employed to test *P. brasiliensis AMY1* expression, so constructions were built using either 889 bp of the strong *H. capsulatum CBP1* promoter (pEC87) or 1950 bp of the promoter region of *H. capsulatum AMY1* (pEC90). Transformation of *H. capsulatum amy1* mutant with either vector pEC87 or pEC90, restored the wild-type “rough” colony morphology (Figure 4, colony morphology), suggesting that in *P. brasiliensis AMY1* is likely to have the same function as in *H. capsulatum*.

Cell wall α-(1,3)-glucan immunofluorescence indicated that wild-type *H. capsulatum* yeasts were intensively stained (Figure 4b–e); this fluorescence was lacking in *ags1*-mutant yeasts (Figure 4g–j) and only present as faint and irregular staining in *amy1*-mutant yeasts (Figure 4l–o). In contrast, *H. capsulatum amy1*-mutant yeasts complemented with either pEC87 (Figure 4q–t) or pEC90 (Figure 4v–y) stained with an intensity similar to that of the wild-type. In addition, quantitative analysis of cell wall α-(1,3)-glucan (that is, the alkali-soluble, acid-precipitable cell wall fraction) indicated that its amount was similar in wild-type *H. capsulatum* yeasts and the *amy1*-null yeasts complemented with *P. brasiliensis AMY1*, while mutant strains (*ags1* and *amy1*) decreased their amount of α-(1,3)-glucan (Table 3). Complementation of *H. capsulatum amy1*-mutant with the *P. brasiliensis AMY1*, suggests a role of *P. brasiliensis* Amyp in the presence and distribution of the cell wall α-(1,3)-glucan.

**Table 3:** Quantification of α-(1,3)-glucan in *H. capsulatum* yeast cells by anthrone assay.

Strain	Relative glucosepresent in F2 (%)
Parent+vector	100±3.34
*ags1-Δ*+vector	2.69±0.51
*amy1-Δ*+vector	13.36±1.31
*amy1-Δ*+pEC87	75.38±1.15
*amy1-Δ*+pEC90	72.11±1.78

a Samples were normalized by preparing a fraction 2 aqueous solution (1 µg ul^−1^).

b Data represent the average from triplicate samples ± standard deviation.

**Figure 5:**
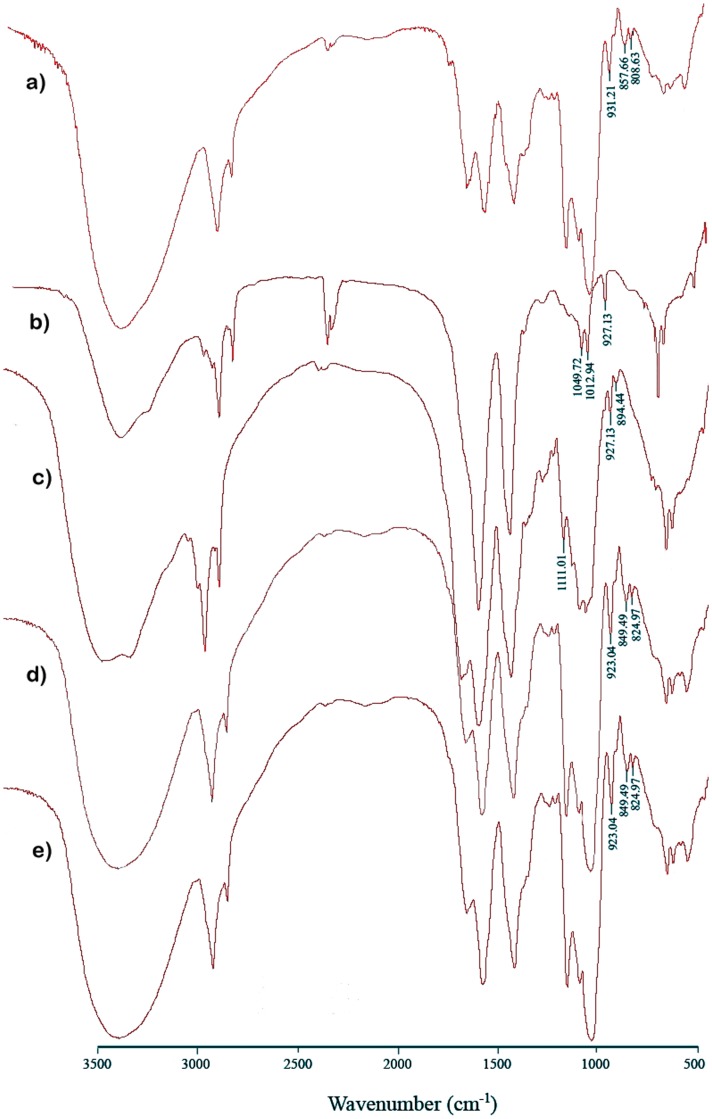
FT-IR spectra of cell wall fraction 2 from *Histoplasma* yeasts. (**a**) *H. capsulatum* strain wild-type (parent). (**b**) *H. capsulatum* strain *ags1*-Δ. (**c**) *H. capsulatum* strain *amy1*-Δ. (**d**) *H. capsulatum* strain *amy1*-Δ complemented with *P. brasiliensis AMY1* gene driven by the *H. capsulatum CBP1* promoter. (**e**) *H. capsulatum* strain *amy1*-Δ complemented with the *P. brasiliensis AMY1* gene driven its native promoter. All control strains were transformed with control vector pCR41.

Structural analysis of each α-(1,3)-glucan-containing fraction was done by FTIR spectroscopy. Absorption bands around 850 cm^−1^ indicated the α-configuration of the glucosyl motif [42], [43], revealing the presence of α-(1,3)-glucan in both *H. capsulatum* wild-type and *amy1*-null yeasts complemented with *P. brasiliensis AMY1* (Figures 5a, 5d, and 5e); on the contrary, this band disappeared in both *Histoplasma* mutant strains. Interestingly, in *H. capsulatum amy1*-Δ the band assigned to α-(1,3)-glucan disappeared, and bands at 890, 920 and 1110 cm^−1^, characteristic of β-glucans, particularly β-(1,3)-glucans, were observed [44] (Figure 5c).

**Figure 6:**
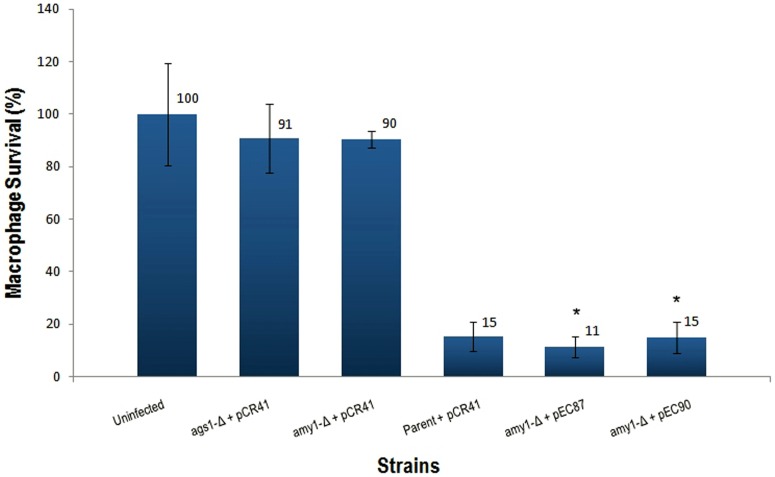
Virulence recovery of *H. capsulatum amy1*-null yeasts complemented with the *P. brasiliensis AMY1* gene. Macrophage survival is measured as the remaining macrophage DNA after incubation of macrophages with *Histoplasma* yeasts. Macrophage DNA remaining at 8 days post infection was normalized to uninfected populations of macrophages. Data represent three independent assays. Error bars represent the standard deviation. (*)Unpaired *t*-tests between *amy1*-Δ+pCR41 and *amy1*-Δ+pEC87 or pEC90; *P*-value <0.0001.

To assess whether virulence was restored in the complemented strains, an *in vitro* virulence assay was carried out by infection of P388D1 macrophage cells with the *H. capsulatum amy1*-Δ strain transformed with either pEC87 or pEC90. The parental and the complemented strains, but not the *H. capsulatum amy1*-null yeast cells, promptly destroyed the macrophage monolayer (Figure 6). This result confirms the requirement of α-1,3-glucan for restoring virulence in *H. capsulatum.*

### Biochemical Properties of *P. brasiliensis* Amy1p

To gain information about some biochemical properties of *P. brasiliensis* Amy1p, we overproduced it in *E. coli* and purified it under native conditions using Ni-NTA agarose. Production of Amy1p in *E. coli* was optimized when the bacteria were grown at 28°C, (maximum yield of 2.4 mg ml^−1^) and subsequently verified with a Western blot (Figures S1a, S1b and S2). Proteins in the Amy1p Ni-NTA-purified sample (calculated mass 64.9 kDa, based on the western profile, Figures S1b and S2) and the negative control were separated by SDS-PAGE and assessed for α-amylase activity by a zymogram. Two faint activity spots were detected in the Amy1p sample, while none was detected in the control (Figure S1c); this pattern was also shown in *A. niger* AmyD and suggested to represent a different protein folding where the lower band seems to be a more active form of the protein [24].

**Figure 7:**
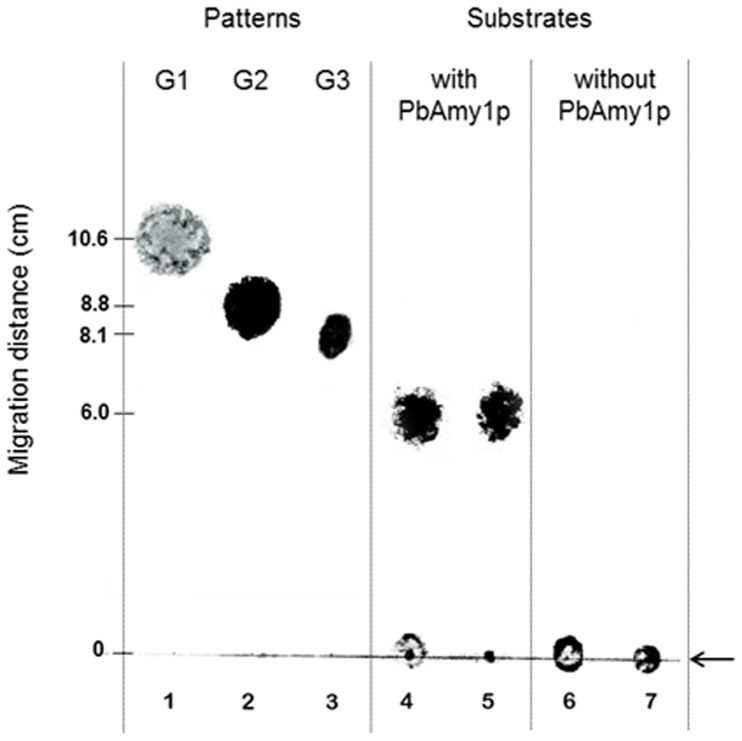
Thin Layer Chromatography (TLC). *P. brasiliensis* Amy1p was incubated for 1 h with amylopeptin and starch. Lanes: 1, glucose (G1); 2, maltose (G2); 3, maltotriose (G3); 4, Amy1p incubated with amylopeptin; 5, Amy1p incubated with starch; 6, amylopeptin; 7, starch. The black arrow at the right down side indicates the loading spots.

Analysis of hydrolysis at different pH values with starch as substrate had an optimum pH ranged in a broad zone from pH 4.0 to 7.0, with a maximum at 5.0 (data not shown). To check for activity towards substrates other than starch, the Ni-NTA-purified Amy1p was also incubated with amylopeptin. This showed that Amy1p had a higher specific hydrolytic activity (µml reducing ends mg^−1^ min^−1^) towards amylopeptin (1.1×10^−2^±0.001) than starch (2.8×10^−3^±0.002). Hydrolyzing activity in the negative control was detected and considered for calculations. Analysis of the reactions by TLC revealed that the product formed from starch and amylopectin were maltooligosaccharides with four or five anhydroglucose units (Figure 7), suggesting that *P. brasiliensis* Amy1p function is related to the production of short oligosaccharides.

## Discussion

Due to the polyspecificity of the GH13 family, these glucosyl hydrolases evolved in such a way that their classification led to no less than 26 different Enzyme Classification (EC) numbers. In order to establish robust groups with an improved correlation between sequence and enzymatic specificity, a further division into 35 subfamilies has been generated [45]. According to it, intracellular fungal α-amylases are classified into group GH13_5, a subfamily previously thought to contain only bacterial α-amylases, while the extracellular α-amylases are members of the GH13_1 subfamily. *In silico* sequence studies of *P. brasiliensis* Amy1p clearly allowed its identification as a member of the GH13_5 subfamily. *P. brasiliensis* Amy1p does not associate with extracellular α-amylases belonging to the GH13_1 subfamily, where *A. oryzae* α-amylase A TAKA is a representative member; instead, it is closely related to two intracellular α-amylases belonging to the *P. brasiliensis* complex (GenBank EEH50612, EEH15936), *P. lutzii* (GenBank XP_002792620) and *H. capsulatum* Amy1p. No amino acids related to α-(1,6)-glycosidase or α-glucanotransferase activities were found in its primary sequence (as reviewed by [18]). A single motif for glycosylation and multiple sites for phosphorylation and myristoylation were identified, indicating possible post-translational modifications of this protein. On the other hand, in agreement with [23], *AMY1* homologues were only found in the genome databases of fungi containing α-(1,3)-glucan in their cell walls (data not shown; http://www.broad.mit.edu/).

Recent articles describing the vesicle and vesicle-free extracellular proteome of *P. brasiliensis* [46] and extracellular proteome of *H. capsulatum* pathogenic phase [47], do not report any α-amylase; which could be explained due to the intracellular nature of the Amy1p reported by [23] and here described. However, previous studies based on the *P. brasiliensis* transcryptome showed an extracellular α-amylase from *P. brasiliensis*, isolate Pb01 (*P. lutzii*), with enzymatic activity on starch but only in mycelial cultures [48]. This activity corresponds to the extracellular α-amylases from *P. lutzii* (Pb01b and Pb01c, Figure 1), supporting the genetic divergence reported by [49].

Transcriptional modulation analysis during the mycelia to yeast transition allows the identification of genes that are over-expressed or dynamically regulated throughout the process. In *P. brasiliensis*, the dimorphic transition is closely related to both pathogenicity and changes in cell wall composition. When the fungus in its mycelial phase turns into yeast, there is a relevant increase in the chitin content of its cell wall, followed by a substitution of the β-(1,3)-glucan by α-(1,3)-glucan [50]. The latter is located as the outermost layer of the fungal cell wall of *P. brasiliensis* and *H. capsulatum* yeast cells [9], [51], and has been found to contribute to pathogenesis in *H. capsulatum* by concealing immunostimulatory β-glucans from detection by host phagocytic cells [51]. The higher level of *P. brasiliensis AMY1* transcription at the yeast phase (Figure 3a) correlates with the presence of α-(1,3)-glucan as the major yeast cell wall polysaccharide and also with a higher expression of *AGS1* in the pathogenic yeast phase of *P. brasiliensis* [10]. Throughout the dimorphic transition from mycelia to yeast, *P. brasiliensis AGS1* transcriptional levels present a gradual increase with higher transcript levels after 48 h [10]. However, during the M-to-Y transition, *P. brasiliensis AMY1* transcriptional levels increased at earlier time points, presenting a sharp decrease at 24 h, only to regain a gradual increase up to 72 h into the M-to-Y transition (Figure 3b). An explanation to this behavior would be that at earlier stages into this transition, Amy1p could be contributing to the building up of a pool of oligosaccharides (as suggested by the TLC results, Figure 7), which could be needed to act as primers for the initiation of the synthesis of α-(1,3)-glucan. Later on, once the oligosaccharides pool required for the initiation of α-1,3-glucan synthesis has been build up, the transcriptional levels of *AMY1* would fall down to basal levels, required for the maintenance of the oligosaccharides pool needed for the synthesis of α-1,3-glucan during growth of the fungus in its yeast phase. However, the later decrease of *AMY1* transcriptional levels at time point 96 h, could be explained by the fact that after 72 h of growth, the *P. brasiliensis* culture is reaching its stationary phase [52]. Therefore, the culture could be overgrowth at 96 h, and the drop in the level of transcription at that time point could be an artifact.

Complementation of *H. capsulatum amy1* mutant by *P. brasiliensis AMY1* successfully restored the rough colony morphology of the parental strain, linked to the presence of cell wall α-(1,3)-glucan. This phenotype was confirmed by molecular, biochemical and virulence tests. As expected, the transcriptional levels of *P. brasiliensis AMY1* under the *CBP1* promoter, a stronger promoter, were 4.5 times higher than those generated with the *AMY1* promoter (data not shown); there were no differences among the data obtained with the two different telomeric vectors indicating that the strength of the *CBP1* promoter did not alter any data. Chemical cell wall analyses and immunofluorescence assays suggested that other cell wall components such as mannoproteins and β-(1,3)-glucan, particularly alkali-soluble β-(1,3)-glucan related to the cell wall flexibility [53], may compensate for the loss of α-(1,3)-glucan as a mechanism to preserve cell wall integrity [54], [55].

The biochemical profile of *P. brasiliensis AMY1* showed a low specific hydrolytic activity that might be explain due to: i) requirement of possible post-translational modifications (glycosylation, phosphorylation, myristoylation) that do not take place in *E. coli* and could be important in structural stabilization or function; ii) requirement of unknown cofactors, such as Ca^2+^, NaCl or EDTA, in order to increase its stability or activity; or iii) testing of unnatural substrates, starch and amilopectin may not be the natural substrates. In order to correct for point (i), we tried to express *P. brasiliensis AMY1* in *S. cerevisiae*, which genome does not codify for α-amylase, but its intracellular expression somehow negatively affected the cell viability.

On the basis of the data here presented, we have shown that *P. brasiliensis* Amy1p, member of GH13_5 subfamily, restores the α-(1,3)-glucan production and virulence in a *H. capsulatum amy1* mutant strain and produces oligosaccharides (maltotetraose and maltopentaose). As mentioned before, the role of Amy1 in the synthesis of α-1,3-glucan could be related with the generation of oligosaccharides that might act as primers for the biosynthesis of this polysaccharide by Ags1p, a member of GH13_22 subfamily. However, its exact contribution to the final α-(1,3)-glucan chemical structure in *P. brasiliensis* remains unclear.

An *in silico* analysis of *P. brasiliensis* genome does not seem to show clustering of *agt*, *ags* and *amy* genes. To confirm or rule out whether *P. brasiliensis AMY1* and/or other genes are involved in the α-(1,3)-glucan biosynthesis, further studies such as their genes disruption or knock-down and analysis of the resulting cells are required. As we progress towards disruption of genes in *P. brasiliensis* associated with the synthesis of such a critical virulence factor, we will be able to better explore the physiology of most fungal dimorphic pathogens as a step forward in the search for new drugs designed to be highly effective against and specific for fungi.

## Supporting Information

Figure S1 **SDS-PAGE analysis of** ***P. brasiliensis*** **Amy1p.** Ni-NTA-purified Amy1p and empty *E. coli* expression vector as a negative control (**N**) were separated by SDS-PAGE and stained for the presence of **(a)** proteins after induction with 1 mM IPTG, using silver staining. **(b)** A 6xHis-tag protein, using anti-His antibody by Western blot. **6xHis Ladder**, molecular weight standard as well as a positive control for western blotting **(c)** α-amylase activity using as substrate amylopectine azure and revealing the bands with iodine. White arrows point to clear areas, showing amylase activity. Samples used for this SDS-PAGE were not denatured, therefore the size of the proteins cannot be estimated directly from their position in relation to the prestained molecular weight marker (**M**).
(TIF)

Figure S2 **Western blot of** ***P. brasiliensis*** **Amy1p native purification.** The protein was purified using Ni-NTA agarose and visualized using an anti-His antibody. **6xHis Ladder**, molecular weight standard and positive control for western blotting; **NI**, not induce with IPTG 1 mM; **CL**, cleared lysated; **NB**, not bound; **W1–W2**, washes; **E1–E4**, eluates.
(TIF)

Table S1 GH13 family proteins used for the alignment and construction of the phylogenetic tree.
(DOCX)
