# Supplementary figures and images for: Expression of Paracoccidioides brasiliensis AMY1 in a Histoplasma capsulatum amy1 Mutant, Relates an α-(1,4)-Amylase to Cell Wall α-(1,3)-Glucan Synthesis
Source: PLoS One. 2012 Nov 20;7(11):e50201. doi: 10.1371/journal.pone.0050201 (PMC3502345; doi:10.1371/journal.pone.0050201)

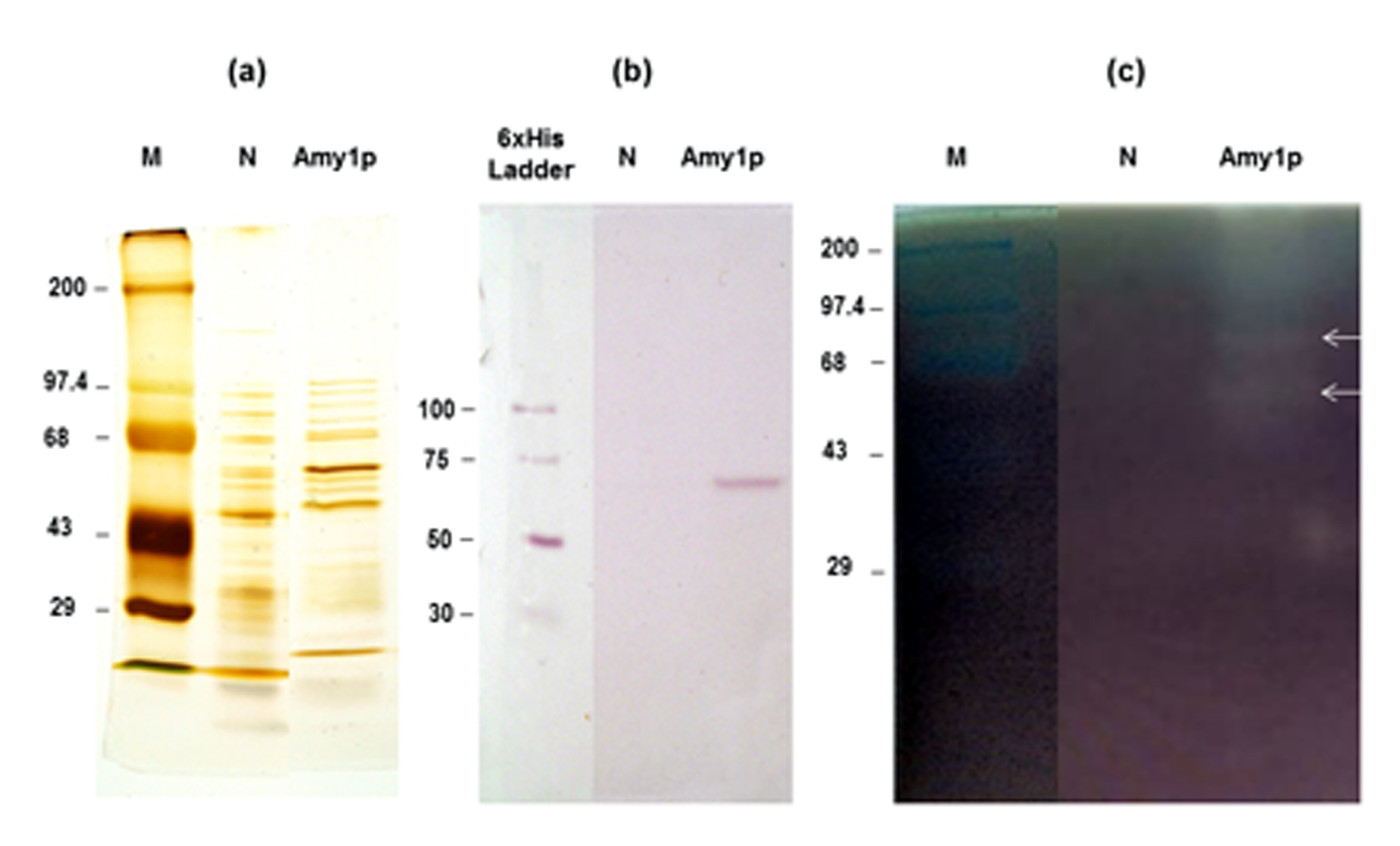

Supplement: Figure S1 — SDS-PAGE analysis of P. brasiliensis Amy1p. Ni-NTA-purified Amy1p and empty E. coli expression vector as a negative control (N) were separated by SDS-PAGE and stained for the presence of (a) proteins after induction with 1 mM IPTG, using silver staining. (b) A 6xHis-tag protein, using anti-His antibody by Western blot. 6xHis Ladder, molecular weight standard as well as a positive control for western blotting (c) α-amylase activity using as substrate amylopectine azure and revealing the bands with iodine. White arrows point to clear areas, showing amylase activity. Samples used for this SDS-PAGE were not denatured, therefore the size of the proteins cannot be estimated directly from their position in relation to the prestained molecular weight marker (M). (TIF) [file pone.0050201.s001.tif]

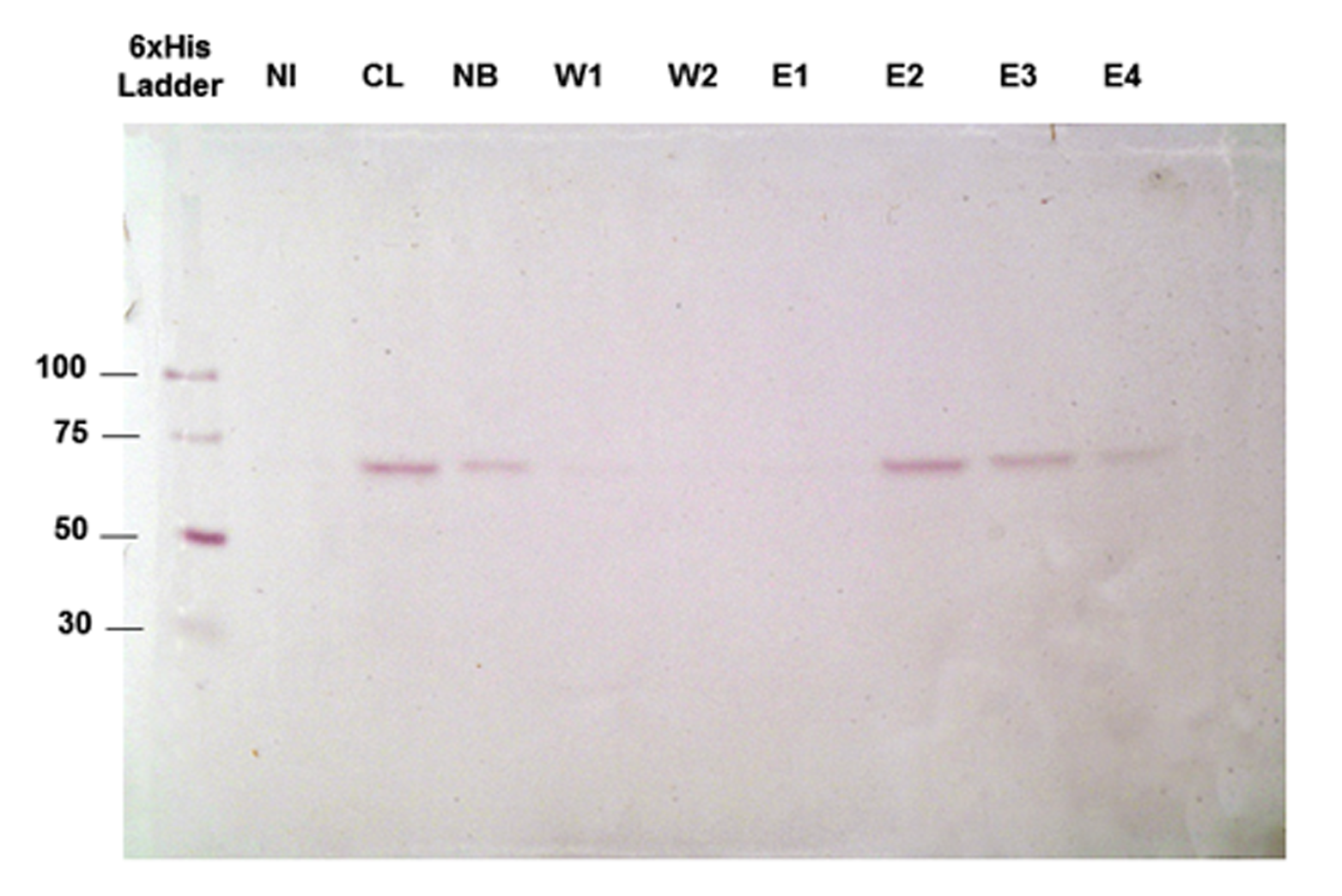

Supplement: Figure S2 — Western blot of P. brasiliensis Amy1p native purification. The protein was purified using Ni-NTA agarose and visualized using an anti-His antibody. 6xHis Ladder, molecular weight standard and positive control for western blotting; NI, not induce with IPTG 1 mM; CL, cleared lysated; NB, not bound; W1–W2, washes; E1–E4, eluates. (TIF) [file pone.0050201.s002.tif]
