# Supplementary material for: Expression of Paracoccidioides brasiliensis AMY1 in a Histoplasma capsulatum amy1 Mutant, Relates an α-(1,4)-Amylase to Cell Wall α-(1,3)-Glucan Synthesis
Source: PLoS One. 2012 Nov 20;7(11):e50201. doi: 10.1371/journal.pone.0050201 (PMC3502345; doi:10.1371/journal.pone.0050201)
Supplement: Table S1 — GH13 family proteins used for the alignment and construction of the phylogenetic tree. (DOCX) [file pone.0050201.s003.docx]

| **Abbreviation** | **Source** | **GenBank accesión number** | **GH13 subfamily** |
| --- | --- | --- | --- |
|  |  |  |  |
|  | **Animals** |  |  |
| Aedaeg | *Aedes aegypti* | AAB60934.1 | 15 |
| Homsap | *Homo sapiens* | AAA52279.1 | 24 |
| Ratnor | *Rattus norvegicus* | BAB39466.1 | 24 |
|  | **Archaea** |  |  |
| Thethi | *Thermococcus thioreducens* | AAT11125.1 | 7 |
| Pyrfur | *Pyrococcus furiosus* | AAB67705.1 | 7 |
|  | **Bacteria** |  |  |
| Cloace | *Clostridium acetobutylicum* | AAD47072.1 | 28 |
| Baclic | *Bacillus licheniformis* | CAA26981.1 | 5 |
| Esccol | *Escherichia coli* | AAN82828.1 | 19 |
| Geoste | *Geobacillus stearothermophilus* | AAA22235.2 | 5 |
| Psehal | *Pseudoalteromonas haloplanktis* | CAA41481.1 | Unknown |
| Saltyp | *Salmonella typhimurium* | AAL22523.1 | 19 |
| Stralb | *Streptomyces albus* | AAA96317.1 | 32 |
| Thecur | *Thermomonospora curvata* | CAA41881.1 | 32 |
| Xancam | *Xanthomonas campestris* | AAA27591.1 | 27 |
| Yerpes | *Yersinia pestis* | AAM87640.1 | 19 |
| SacdeB | *Saccharophagus degradans* | ABD79837.1 | 19 |
| SacdeP | *Saccharophagus degradans* | ABD79827.1 | 6 |
| SacdeA | *Saccharophagus degradans* | ABD82195.1 | Unknown |
| Strmut | *Streptococcus mutans* | AAC35010.1 | 5 |
|  | **Fungi and yeasts (fam GH13_1)** |  |  |
| Emenid | *Aspergillus nidulans* | AAF17100.1 | 1 |
| Aspnig-AmyC | *Aspergillus niger* | CAK44693.1 | 1 |
| Aspory-TAA | *Aspergillus oryzae* | AAA32708.1 | 1 |
| CryptoS2 | *Cryptococcus sp. S-2* | BAA12010.1 | 1 |
| Pichbu | *Pichia burtonii* | BAF98616.1 | 1 |
| Sacfib | *Saccharomycopsis fibuligera* | CAA29233.1 | 1 |
| Pb01b | *Paracoccidioides brasiliensis* Pb01 | XP_002795211.1 |  |
| Pb01c | *Paracoccidioides brasiliensis* Pb01 | XP_002791218.1 | Unknown |
|  | **Fungi (fam GH13_5)** |  |  |
| Aspnig-AmyD | *Aspergillus niger* | CAK37367.1 | 5 |
| Aspnig-AmyE | *Aspergillus niger* | CAK40250.1 | 5 |
| Aspnid1 | *Aspergillus nidulans* | EAA63277.1 | 5 |
| Aspory1 | *Aspergillus oryzae* | BAE56147.1 | 5 |
| Cryneo | *Cryptococcus neoformans var. neoformans* | AAW44866.1 | 5 |
| Hcap | *Histoplasma capsulatum* | ABK62854.1 | 5 |
| Maggri1 | *Magnaporthe grisea* | XP_364319.2 | 5 |
| Maggri2 | *Magnaporthe grisea* | XP_364797.2 | 5 |
| Maggri3 | *Magnaporthe grisea* | XP_360744.1 | 5 |
| Neucra | *Neurospora crassa* | CAE75731.1 | 5 |
| Pb73 | *Paracoccidioides brasiliensis* IVIC Pb73 / ATCC 3207 | ABS11196.1 | 5 |
| Podans | *Podospora anserina* | CAP70916.1 | 5 |
| Pb01a | *Paracoccidioides brasiliensis* Pb01 (cytoplasmatic) | XP_002792620.1 | ? |
| Pb18 | *Paracoccidioides brasiliensis* Pb18 | EEH50612 | Unknown |
| Pb03 | *Paracoccidioides brasiliensis* Pb03 | EEH15936 | Unknown |
|  | **Plants** |  |  |
| Orysat | *Oryza sativa* (rice) | AAA33885.1 | 6 |
| Zeamay | *Zea mays* (corn) | AAA50161.1 | 6 |
| Phavul | *Phaseolus vulgaris* (kidney bean) | BAA33879.1 | 6 |

**TablaS2**
